# Inflammatory myofibroblastic tumor presenting as ileocolic intussusception: A case report

**DOI:** 10.1002/ccr3.8064

**Published:** 2023-10-23

**Authors:** Nischal Khanal, Rupak Subedi, Nirajan Shrestha, Shristi Shrestha

**Affiliations:** ^1^ General Surgery Madan Bhandari Academy of Health Sciences Hetauda Nepal; ^2^ Madan Bhandari Academy of Health Sciences Hetauda Nepal

**Keywords:** abdominal pain, hemicolectomy, inflammatory myofibroblastic tumor, intussusception, spindle cell proliferation, vomiting

## Abstract

**Key Clinical Message:**

The report urges considering rare neoplastic tumor, like IMT, in intussusception cases and underscores the vital role of comprehensive and swift diagnosis in influencing treatment choices and patient results.

**Abstract:**

We present a case of a 36‐year‐old male who presented with abdominal pain and vomiting. Inflammatory markers were elevated in routine investigations, while other laboratory parameters were within normal limits. Ultrasound imaging revealed a target lesion, which required further evaluation with a CT scan and confirmed ileocolic intussusception. An exploratory laparotomy showed a solid lesion measuring 5 by 6 cm in the cecum, along with evidence of ileocolic intussusception. A standard right hemicolectomy was performed, and the postoperative course was uneventful. Histopathological examination showed an inflammatory myofibroblastic tumor with nodular hyperplasia. The report highlighted the importance of assessing rare neoplastic causes in patients with intussusception.

## Graphical abstract

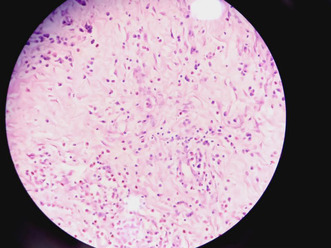

## 1 INTRODUCTION

Inflammatory myofibroblastic tumors (IMTs) are rare neoplasms characterized by proliferative myofibroblastic spindle cells accompanied by a prominent inflammatory infiltrate.^1^ IMTs predominantly affect children and young adults but can occur at any age.^2^ The most common site of occurrence is the lung, followed by the extrapulmonary sites like liver, pancreas, intestine, and bones.^3^ Intestinal involvement is relatively rare, and ileocolic intussusception caused by IMT is an even rarer presentation.^4^ We report a case of a 36‐year‐old male who presented with abdominal pain, vomiting, and subsequent diagnosis of IMT leading to ileocolic intussusception.

## 2 CASE PRESENTATION

A 36‐year‐old male presented to the emergency department with a three‐day history of diffuse abdominal pain, followed by vomiting. The pain started from the right lower quadrant and later generalized. On physical examination, mild tenderness was present on deep palpation in the right lower quadrant of the abdomen. The patient had no significant medical history and denied any recent weight loss or changes in bowel habits.

**FIGURE 1:**
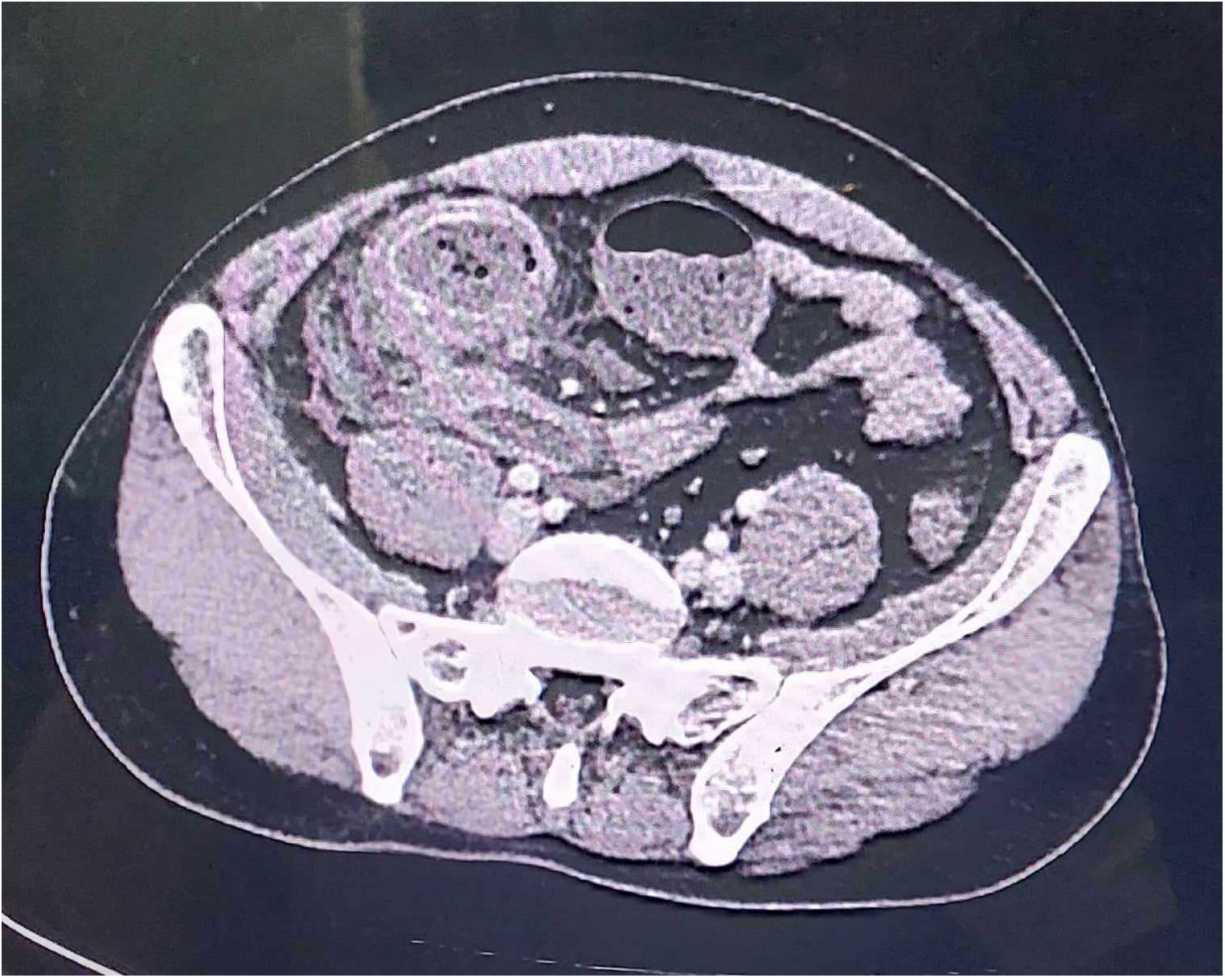
Axial image showing thickened ileocolic junction with target stratification pattern.

**FIGURE 2:**
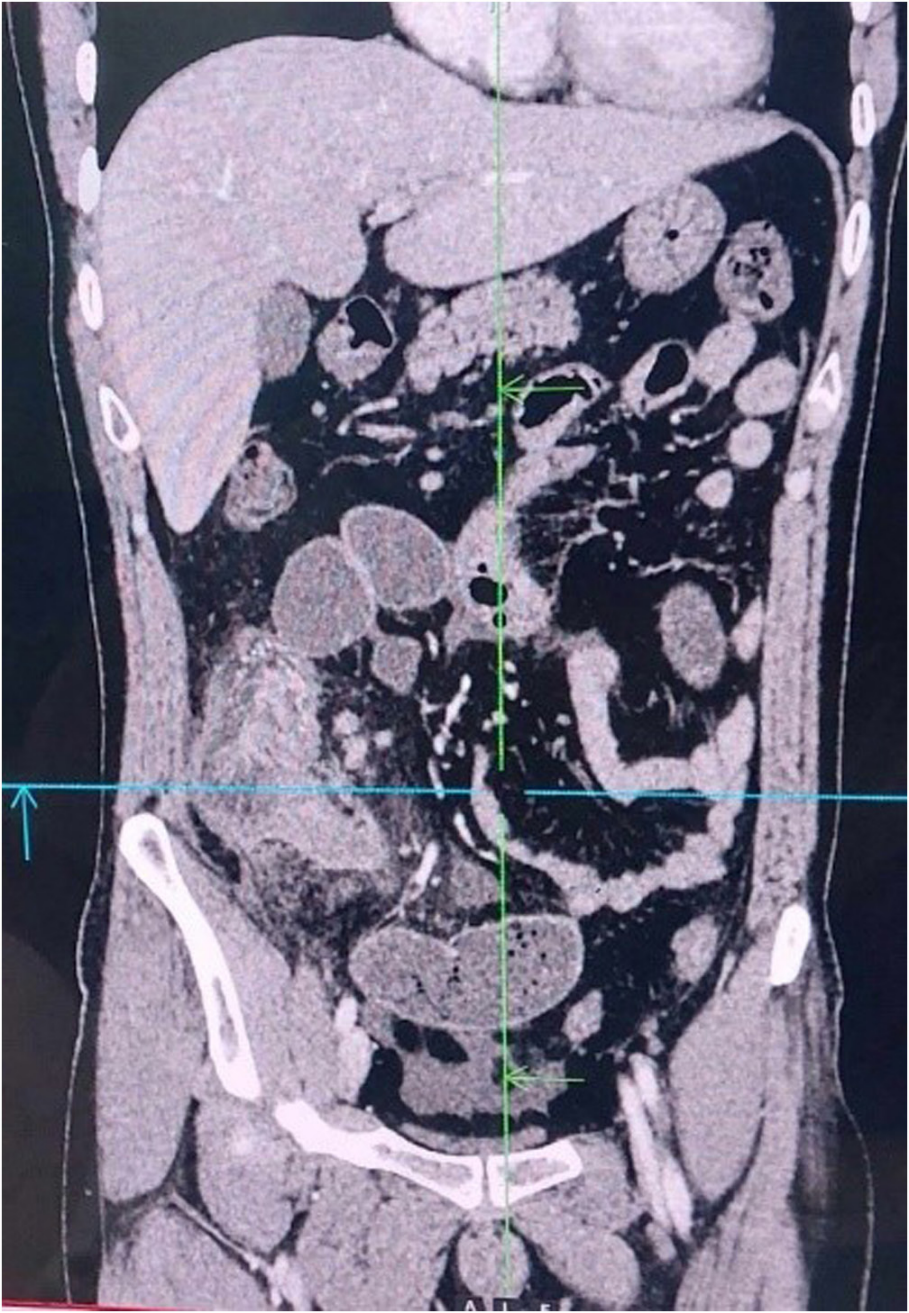
Coronal image showing intraluminal pseudomass appearance with perlesional fat strandings.

Laboratory investigations revealed raised inflammatory marker. A complete blood count revealed elevated WBCs (17,100/μL) with neutrophilia (80%). Blood urea nitrogen and creatinine level were normal and other hematological and biochemical parameters were within normal limits. An ultrasound examination of the abdomen showed a target lesion in the right lower quadrant, raising the suspicion of intussusception. A subsequent CT scan confirmed segmental, circumferential thickening of the terminal ileocolic junction showing a target stratification pattern (Figure 1). The protrusion of terminal ileum into ascending colon gave an intraluminal pseudomass appearance (Figure 2). The small bowel loops proximal to ileocolic junction was dilated with outer‐to‐outer diameter of 3.2 cm and mottled appearance giving small bowel feces sign.

**FIGURE 3:**
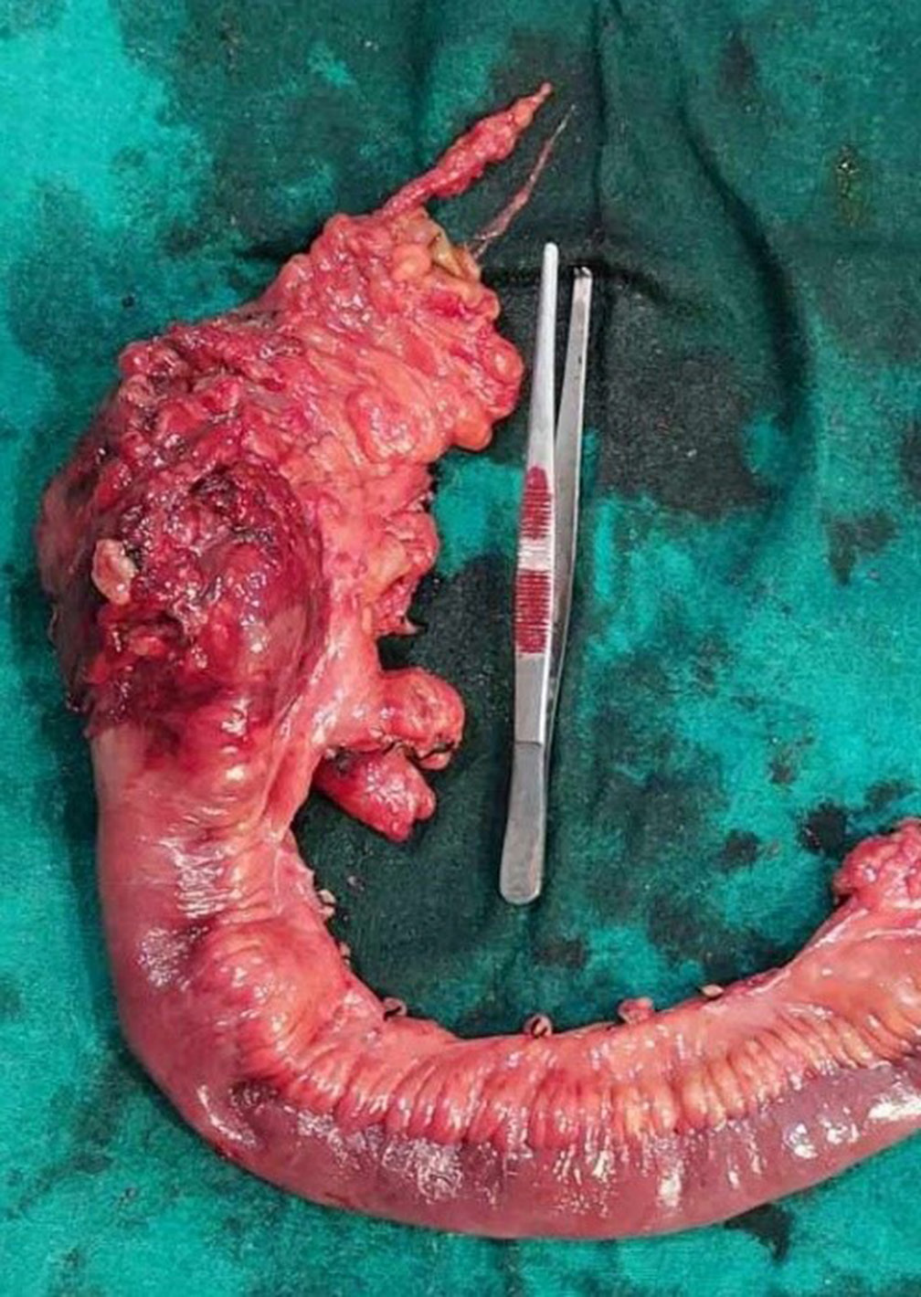
Tumor mass removed along with segment of ileum.

**FIGURE 4:**
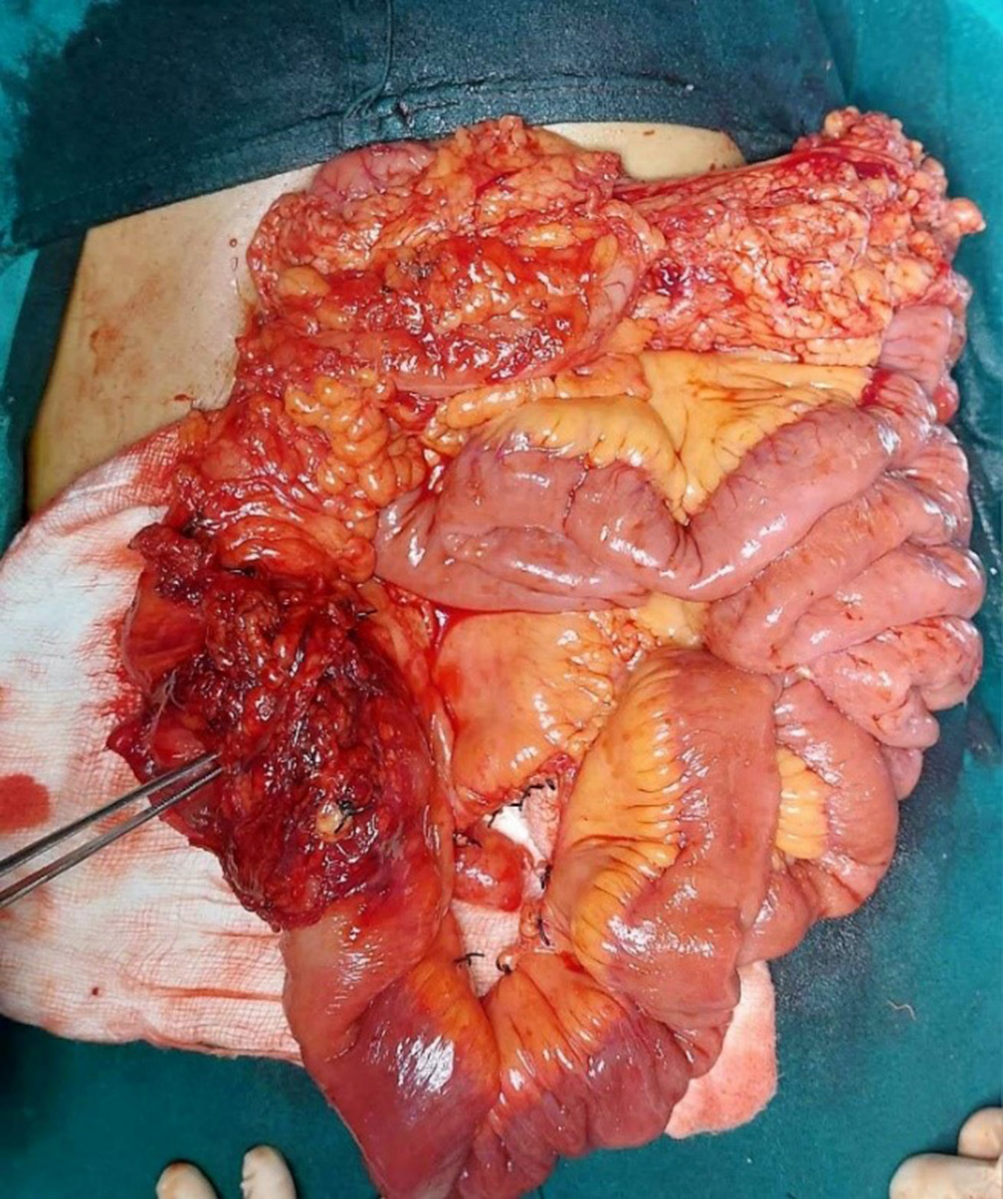
Intraoperative image with tumor insitu.

The patient underwent an exploratory laparotomy. Intraoperatively there was a solid lesion measuring approximately 5 × 6 cm, well‐demarcated smooth mass involving the cecum, approximately 2 cm proximal to ileocolic junction (Figures 3, 4). There was no evidence of ascites, no mesenteric lymphadenopathy and liver surface appeared normal. Lead point of ileocolic intussusception was the mass and right sided standard hemicolectomy performed. On cut surface, the lesion had diffuse gray to yellowish appearance. The procedure was completed without complications, and the specimen was marked and sent for histopathological examination.

The postoperative course was unremarkable, and after 24 h of observation in intensive care unit (ICU) the patient was transferred to the surgery ward. He recovered well and discharged after fifth postoperative day.

**FIGURE 5:**
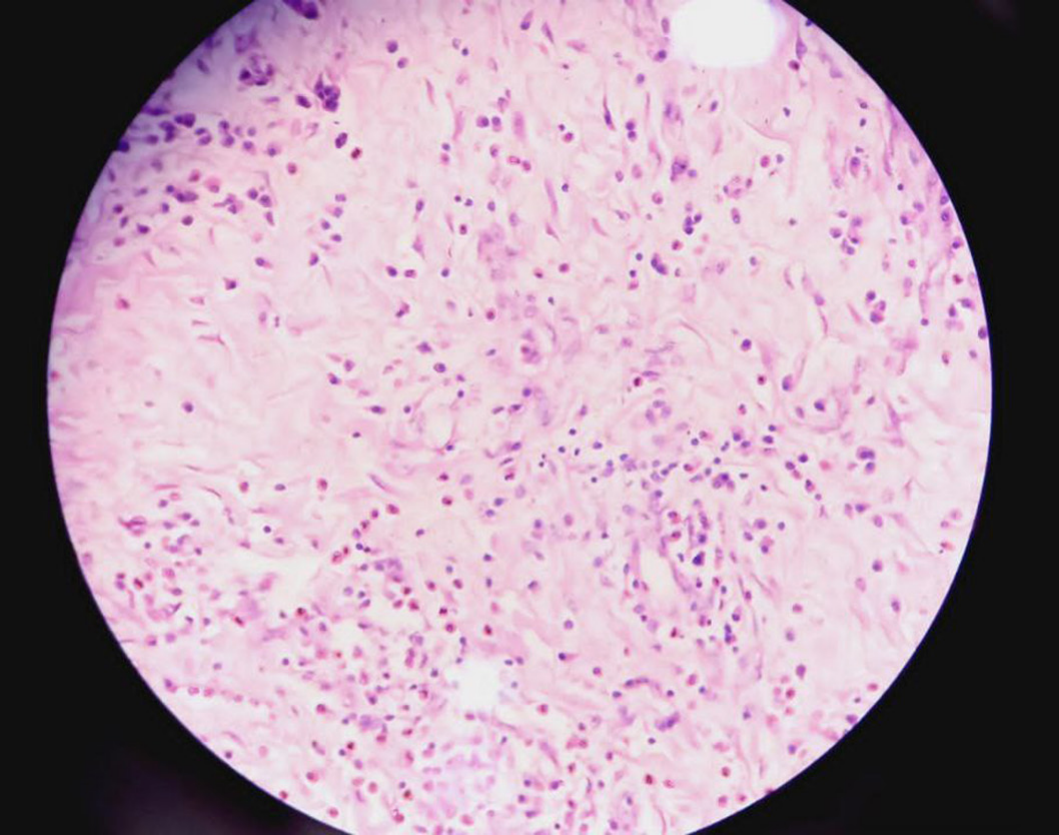
Singly scattered spindle fibroblastic cells along with stellate cells and ganglion like polygonal cells intermingled with acute and chronic inflammatory cells.

**FIGURE 6:**
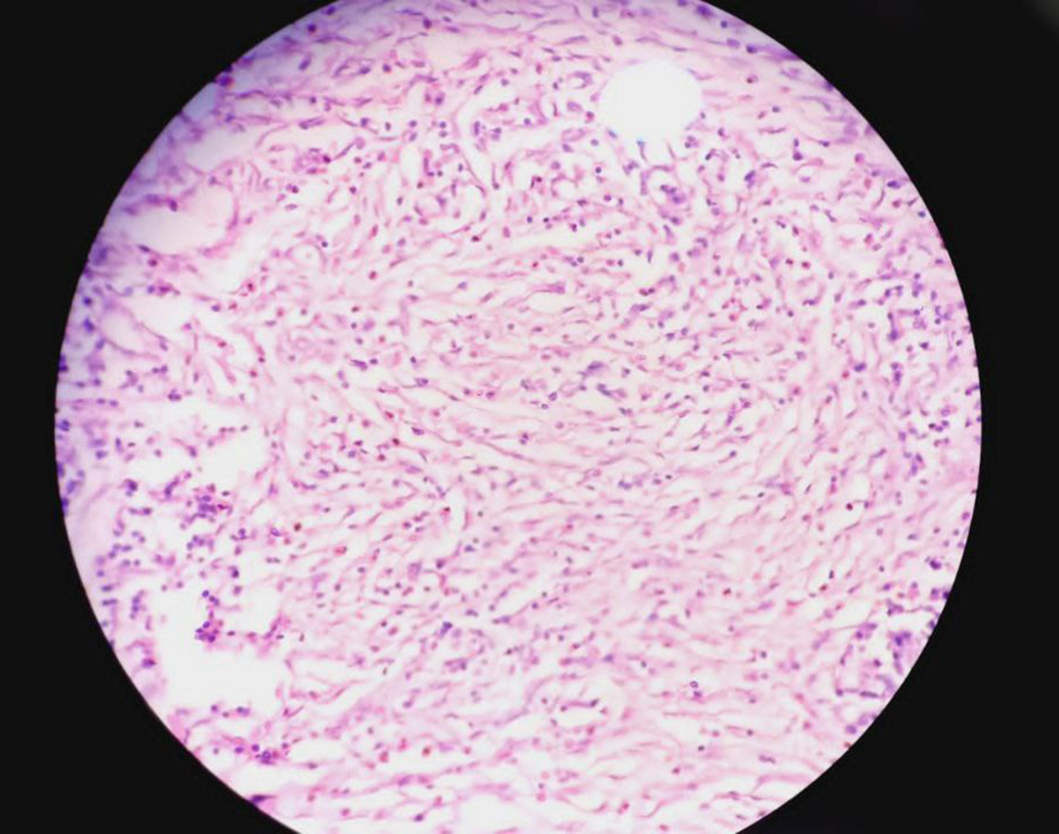
Spindle cells arranged in storiform pattern indispersed with moderate amount of acute inflammatory cells composed of lymphocytes plasma cells and eosinophils.

Histopathological examination of the resected specimen showed an inflammatory myofibroblastic tumor. The tumor showed nodular hyperplasia with diffuse eosinophilic infiltrate, subserosal bland spindle to stellate cells proliferation along with lymphoid aggregates, lymphocytes, plasma cells, and eosinophils. (Figures 5, 6).

## 3 DISCUSSION

Inflammatory myofibroblastic tumors being rare neoplasms with diverse clinical presentations can affect various organs like lungs, liver, endolarynx, maxillary sinus, oral cavity etc.^5^ However, their involvement in the gastrointestinal tract is particularly rare and their expression as intussusception is even rarer.^4^ Their exact etiology remains uncertain but they can even mimic various inflammatory conditions and their behavior ranges from benign to locally aggressive or rarely malignant, making the diagnosis challenging.

Some common causes of adult ileocolic intussusception are adhesions, polyp, inflammatory bowel disease, gastro intestinal tumor (GIST), leiomyosarcoma etc.^6^, ^7^ Inflammatory myofibroblastic tumor presenting as ileocolic intussusception is a rare diagnosis, so it is even difficult for a resources equipped centers to diagnose. Diagnosing this rare condition in Hetauda Hospital, a district hospital located in suburban region with inadequate resources is quite challenging. The diagnosis of this condition at our center either reflects the inadequacies in studies or not frequently reported.

In our case, the patient's presentation with abdominal pain, vomiting, and imaging findings of intussusception prompted further investigation, leading to the diagnosis of an IMT. Surgical resection followed by histopathological examination of the resected specimen performed.

Immuno histochemical staining (IHC) which helps to identify specific marker expressed by tumor cells like smooth muscle actin (SMA), anaplastic lymphoma kinase (ALK Protein), vimentin, and CD34. These IHC features can help in distinguishing benign or malignant nature of the tumor. Together in conjunction with histopathological features, they can help to establish confirmatory diagnosis. However, in suburban area like ours, patient presents with their complaints and after getting relief from their issues, they do not come up to hospital for follow up. Therefore, in our case we are unable to give immunohistochemistry (IHC) reports as he was lost to follow up.

Further studies, research and case reports are necessary to understand the etiology, behavior, and optimal management strategies for IMTs, particularly those involving gastrointestinal tract. Additionally, increased awareness among clinicians is also essential to ensure early recognition, prompt diagnosis, and appropriate surgical intervention, as demonstrated in this case, are crucial for optimal management and favorable patient outcomes of these rare tumors.

## 4 CONCLUSION

This case report shares a very rare condition of inflammatory myofibroblastic tumor (IMT), presenting with ileocolic intussusception in a middle‐aged man, which is extremely uncommon and can be confused with other inflammatory conditions; however, through diagnostic evaluation and multidisciplinary approach can result in early diagnosis, optimal management, and favorable patient outcomes of these rare tumors.

## AUTHOR CONTRIBUTIONS

**Nischal Khanal:** Conceptualization; data curation; formal analysis; resources; supervision. **Rupak Subedi:** Data curation; formal analysis; investigation; software; writing – review and editing. **NIrajan Shrestha:** Conceptualization; project administration; resources; supervision. **Shristi Shrestha:** Conceptualization; resources; writing – review and editing.

## CONFLICT OF INTEREST STATEMENT

There are no conflict of interest regarding this manuscript.

## ETHICS STATEMENT

Please note that due to patient confidentiality and privacy considerations, access to specific clinical information may be subject to approval by our institution's ethics committee and may require compliance with relevant data protection regulations.

## CONSENT

Written informed consent was obtained from the patient to publish this report in accordance with the journal's patient consent policy.

## Data Availability

The data supporting the findings of this case report are available upon request from the corresponding author. Requests for data access should be directed to Dr Nischal Khanal at nischal.np@gmail.com.
